# Antiprotozoal Activity of *Achillea ptarmica* (Asteraceae) and Its Main Alkamide Constituents †

**DOI:** 10.3390/molecules19056428

**Published:** 2014-05-20

**Authors:** Julia B. Althaus, Marcel Kaiser, Reto Brun, Thomas J. Schmidt

**Affiliations:** 1 Institut für Pharmazeutische Biologie und Phytochemie (IPBP), University of Münster, PharmaCampus, Corrensstraße 48, Münster D-48149, Germany; E-Mail: julia.althaus@uni-muenster.de; 2 Swiss Tropical and Public Health Institute (Swiss TPH), Socinstraße 57, Basel CH-4002, Switzerland; E-Mails: Marcel.Kaiser@unibas.ch (M.K.); Reto.Brun@unibas.ch (R.B.); 3 University of Basel, Petersplatz 1, Basel CH-4003, Switzerland

**Keywords:** *Achillea ptarmica*, antiprotozoal activity, alkamide, *Plasmodium falciparum*, *Trypanosoma brucei rhodesiense*, *Trypanosoma cruzi*, *Leishmania donovani*

## Abstract

In the course of our ongoing screening of plants of the family Asteraceae for antiprotozoal activity, a CH_2_Cl_2_-extract from the flowering aerial parts of *Achillea ptarmica* L. (sneezewort yarrow) was found to be active *in vitro* against *Trypanosoma brucei rhodesiense* (IC_50_ = 0.67 µg/mL) and *Plasmodium falciparum* (IC_50_ = 6.6 μg/mL). Bioassay guided fractionation led to the isolation and identification of five alkamides from the most active fractions. Pellitorine and 8,9-*Z*-dehyropellitorine are the main components of the extract. Beside these olefinic acid amides, four alkamides with diene-diyne structures were isolated. All alkamides were tested for antiprotozoal activity *in vitro*. Pellitorine was the most active compound so far within this study against *P. falciparum* (IC_50_ = 3.3 µg/mL), while 8,9-*Z*-dehydropellitorine was most active against *T. b. rhodesiense* (IC_50_ = 2.0 µg/mL). The activity of pure pellitorine against *Plasmodium* is higher than that of the crude extract and thus explains the activity of the latter. None of the isolated alkamides, however, was as active against *T. b. rhodesiense* as the crude extract whose antitrypanosomal activity must therfore be due to a synergistic effect of the isolated compounds or to more active yet to be identified constituents.

## 1. Introduction

Plants of the Asteraceae (sunflower) family are one of the main focuses in our search for natural products with activity against the major protozoan pathogens responsible for human diseases, *i.e.*, *Plasmodium*, *Trypanosoma* and *Leishmania* species [1,2,3,4,5,6,7]. In this screening, plants from this family have repeatedly yielded natural compounds with interesting activity against these parasites, especially *T. brucei rhodesiense* (*Tbr*), the causative agent of the East African form of human trypanosomiasis (sleeping sickness). Most of these compounds belong to the characteristic class of sesquiterpene lactones [4,5], but we have also reported on antiprotozoal activity of some flavonoids and a chromene derivative from the Asteraceae [6,7]. Generally, IC_50_ values < 1 µg/mL against *Tbr* are considered a promising activity level for crude extracts. In the present study, a dichloromethane extract of flowering aerial parts of *Achillea ptarmica* L. (sneezewort yarrow) was found to display interesting anti-*Tbr* activity with an IC_50_ of 0.67 µg/mL and relatively moderate cytotoxicity. Furthermore, it also displayed moderate antiplasmodial activity against the etiologic agent of tropical malaria, *P. falciparum* (*Pf*, IC_50_ 6.6 µg/mL).

*A. ptarmica*, unlike its relative *A. millefolium*, has not been reported to contain sesquiterpene lactones. Its main constituents, besides some essential oil [8] and flavonoids [9], are known to be alkylamides (“alkamides”), *i.e.*, carboxamides of olefinic and polyynic carboxylic acids with various amine components [10,11]. Such carboxamides have previously been reported to possess local anaesthetic and insecticidal [10,12] as well as anti-inflammatory and immunomodulating [13] activities. The major aim of this work was therefore to identify the chemical constituents responsible for the antiprotozoal activity.

## 2. Results and Discussion

### 2.1. Antiprotozoal Activity of Achillea Crude Extracts

During our screening of Asteraceae extracts, we investigated, among others, a dichloromethane extract of flowering aerial parts of *Achillea ptarmica* which displayed an interesting level of activity against *Tbr* and moderate activity against *Pf*, while being essentially inactive against *T. cruzi* (*Tcr*; Chagas disease) and *Leishmania donovani* (*Ldo*; visceral leishmaniasis). The biological data are reported in Table 1. It is especially noteworthy that the cytotoxic activity against rat skeletal myoblasts (L6 cell line) used as control cells was over 40 times lower, so that this extract also displayed a favorable selectivity against *Tbr*. Interestingly, an extract of *A. millefolium* (same plant parts, but obtained with diethyl ether) displayed a dissimilar activity pattern with somewhat higher activity against *Pf*, but negligibly low activity against the trypansomatid parasites. The extract of *A. ptarmica* was therefore chosen for a detailed study of its potential antitrypanosomal as well as antiplasmodial constituents.

**Table 1:** *In vitro* antiprotozoal and cytotoxic activity [IC_50_ values in µg/mL; values in µM of pure compounds are reported in brackets] of crude Asteraceae extracts and of the alkamides isolated from *A. ptarmica*. Selectivity indices (SI) represent the ratio of cytotoxic over antiprotozoal IC_50_ values. Data are means of two independent determinations ± deviation from mean value.

	*Tbr*	*Tcr*	*Ldo*	*Pf*	Tox. L6	SI *Tbr*	SI *Pf*
Crude extracts							
***A. ptarmica*** (aerial flowering parts; CH_2_Cl_2_-extract)	0.67 ± 0.35	43.7 ± 8.1	14.9 ± 0.7	6.58 ^a^ ± 1.15	27.9 ± 8.0	41.64	4.24
***A. millefolium*** (aerial flowering parts; Et_2_O-extract)	23.5 ± 1.5	44.7 ± 4.5	6.76 ± 2.51	2.82 ^a^ ± 0.26	41.2 ± 13.0	1.75	14.6
***E. purpurea*** (flowering aerial parts; CH_2_Cl_2_-extract)	3.70 ± 1.71	>10	>10 ^c^	4.39 ± 0.31 ^b^	52.5 ± 2.1	14.2	12.0
***A. pyrethrum*** (roots, CH_2_Cl_2_-extract)	>10 ^c^	8.83 ± 0.75	4.22 ± 1.57	3.04 ^b^ ± 0.07	13.4 ± 3.2	<1.34	4.41
Isolated alkamides							
Pellitorine (**1**)	5.35 ± 0.54 (24.0)	8.45 ± 1.08 (37.9)	5.96 ± 0.16 (26.7)	3.26 ^b^ ± 0.53 (14.6)	45.5 ± 9.1 (201.8)	8.41	13.85
8,9-***Z***-Dehydropellitorine (**2**)	2.00 ± 0.06 (9.1)	14.2 ± 2.5 (64.3)	5.01 ± 0.12 (22.7)	6.48 ^b^ ± 0.55 (29.3)	16.5 ± 0.2 (74.7)	8.25	2.55
(**3**)	6.66 ± 0.22 (29.1)	19.9 ± 1.5 (86.9)	8.87 ± 1.33 (38.7)	5.84 ^b^ ± 0.17 (25.5)	46.1 ± 3.2 (201.3)	6.92	7.89
(**4**+**5**) (3:1 mixture)	3.50 ± 0.44	8.36 ± 2.90	11.8 ± 0.1	6.89 ± 0.21 ^b^	43.4 ± 5.0	12.4	6.30
Anacycline (**6**)	5.12 ± 0.95 (18.9)	42.0 ± 4.5 (154.9)	>100 (>369)	7.23 ± 0.40 ^b^(26.7)	48.7 ± 1.1 (18.0)	9.5	6.74
Positive controls							
Melarsoprol	0.003 ± 0.001 (0.008)						
Benznidazole		0.439 ± 0.094 (1.688)					
Miltefosine			0.127 ± 0.052 (0.312)				
Chloroquine				0.080 ^a^ ± 0.003 (0.250)			
Chloroquine				0.003 ^b^ ± 0.001 (0.009)			
Podophyllotoxin					0.008 ± 0.001 (0.019)		

^a^ K1 strain; ^b^ NF54 strain; ^c^ preliminary data from 2-concentration assay conducted at 2 and 10 µg/mL.

### 2.2. Bioassay-Guided Fractionation of Achillea ptarmica Extract and Isolation of Alkamides

Using a gradient of hexane/ethyl acetate the dichloromethane extract was separated by column chromatography (CC) into 30 fractions which were combined after TLC control (see Experimental Section). All fractions were analyzed by UHPLC/+ESI QTOF MSMS and representative samples chosen for re-evaluation of their antiprotozoal activity. At this stage, each of the chosen fractions was tested for percent growth inhibitory activity at two concentrations (see Table 2).

**Table 2:** *In vitro* growth inhibition [%] at two concentrations of the *A. ptarmica* crude extract and selected subfractions against the various parasites.

	*Tbr*		*Tcr*		*Ldo*		*Pf* (NF54)	
	10 µg/mL	2 µg/mL	10 µg/mL	2 µg/mL	10 µg/mL	2 µg/mL	10 µg/mL	2 µg/mL
Total extract	100	0	1	3.4	19.4	9.4	63.8	6.1
CC fractions								
II	4.3	25	0.6	0	8.9	5.1	5	4.7
IV	7.2	0	0	16.6	27.5	15.3	6	0
Vb	0	25	0	0	17.8	11.8	0	3.9
XVI	45.3	0	21.1	21.9	25.4	9.2	47.1	2.5
XX	66.8	0	18.9	0	17.2	21.7	9.8	0
XXII	90.2	6.4	8.5	0	35.7	8	35.7	7.7
XXIII	99.9	0	4.2	0	32.4	9.1	96.8	3.9
XXV	100	0	3.9	0	53.6	10.1	99.9	13.7
XXVIII	100	9.4	0	0	42.1	10	99.6	11.7

**Figure 1:**
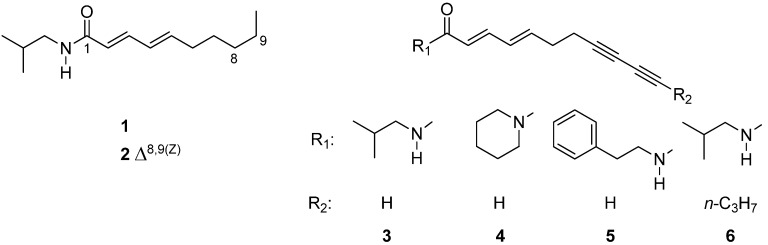
Structures of isolated compounds.

**Figure 2:**
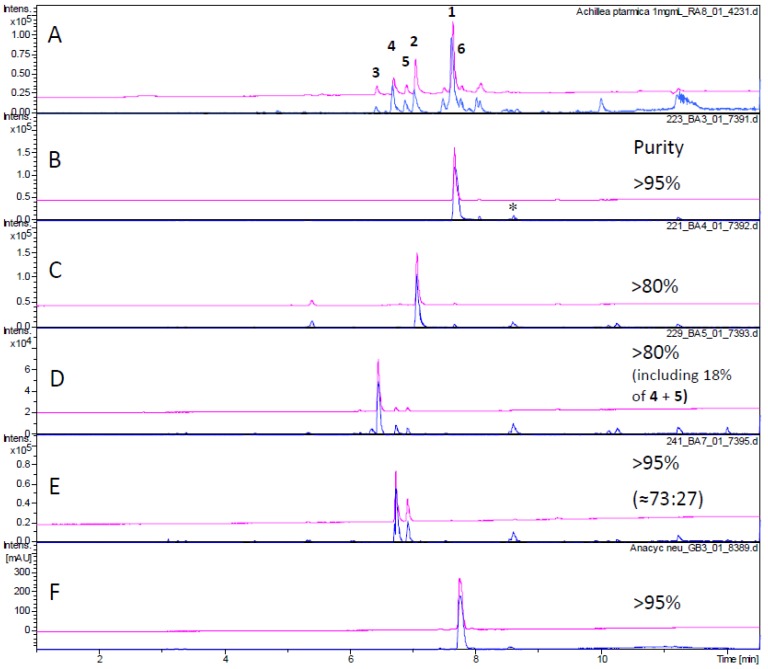
UHPLC/+ESI QTOF MSMS analysis of isolated compounds. Magenta: UV chromatogram at 260 nm; blue: Base peak chromatogram *m/z* 100–500. (**A**) CH_2_Cl_2_ extract of *A. ptarmica* (1 mg/mL); (**B**) pellitorine (**1**); (**C**) 8,9-*Z*-dehydropellitorine (**2**); (**D**) compound **3**; (**E**) compounds **4** + **5**; (**F**) anacycline (**6**); (**B**–**E**) 0.1 mg/mL; * denotes a solvent impurity also appearing in a blank chromatogram.

Obviously, the antitrypanosomal as well as antiplasmodial activity was concentrated in the later fractions. Subsequently, the major constituents were isolated from these fractions or directly neighboring fractions containing the same constituents. Thus, from fractions XXI, XXII, XXIII, XXV and XXVIII, the main alkamides **1**–**6** were isolated (Figure 1). Compounds **4** and **5** could only be obtained as a mixture containing them in a ratio of approximately 3:1. All compounds were unambiguously identified on the grounds of their exact masses as obtained from UHPLC/+ESI QTOF MSMS analyses (see Figure 2) as well as their ^1^H-NMR data, which were in full agreement with literature values [10,11,14].

### 2.3. Antiprotozoal Activity of Isolated Alkamides

The isolated compounds were tested for their antiprotozoal activity (see Table 1). The main constituent, pellitorine (**1**) was found to be the most active compound against *Pf*, with an IC_50_ of 3.26 µg/mL (14.6 µM). The antiplasmodial activity of **1** is thus about 2-fold higher than that of the crude extract and it also presents a higher selectivity index against this parasite than the extract. It can hence be concluded that this compound is mainly responsible for the activity of the latter against *Pf*. It is interesting to note that contradictory information exists in the literature with respect to the antiplasmodial activity of this compound. While pellitorine has been reported inactive up to a concentration of 20 µg/mL against the K1 strain of *Pf* [15], earlier authors reported an IC_50_ of of 5.4 µg/mL for N-isobutyldeca-2,4-diene (double bond geometry not specifically mentioned) against this same strain [16]. Our results therefore confirm the previous finding.

Compound **2** (8,9-*Z*-dehydropellitorine) was found the most active of the isolated alkamides against *Tbr*. Its IC_50_ value of 2.00 µg/mL (9 µM), however, corresponds to a 3-fold lower activity in comparison with the crude extract. Its selectivity index is about 5-fold lower than that of the total extract. Therefore, the promising activity and selectivity of the total extract cannot be attributed to any of the isolated alkamides on its own.

It is hence conceivable that either some minor constituent(s) not hitherto isolated was/were responsible for the high activity of the crude extract, or that some “synergistic” effect was at work. A few additional alkamides could be detected at low concentrations, which we are currently isolating from a larger quantity of the plant material.

### 2.4. Antiprotozoal Activity of Further Alkamide-Containing Plant Species

In order to assess the potential activity of other Asteraceae known to contain alkamides as major chemical constituents, we have performed preliminary tests with extracts of *Echinacea purpurea* (L.) Moench (Purple Coneflower) and of *Anacyclus pyrethrum* (L.) Lag. (Spanish Pellitory), both well known for their alkamide content [12,13,17]. Both were found to exhibit significant activity against *Pf* with IC_50_s < 10 µg/mL. *E. purpurea* showed activity in the same concentration range against *Tbr* while *A. pyrethrum* was distinctly less active against the latter parasite but showed activity at a similar level against *Ldo* (see Table 1). Both species will therefore be included in further studies. It should not remain unmentioned that other authors have recently reported independently on the antiplasmodial activity of an ethyl acetate extract of *A. pyrethrum* roots, without further specification of the active constituents or an IC_50_ value [18]. We report here for the first time that these roots also show activity against *L. donovani*. An extract preparation from aerial parts of *E. purpurea* has previously been described as having antitrypanosomal and antileishmanial activity. However, in this case, an aqueous extract was used and the reported inhibitory concentrations were in the mg/mL concentration range. Moreover, no indications on the chemical compounds responsible for these effects were made [19].

## 3. Experimental

### 3.1. Analytical Procedures and Instrumentation

#### 3.1.1. Preparative High-Performance Liquid Chromatography (HPLC)

All separations were performed on a Waters preparative HPLC system (Waters 515 pumps with a Knauer single wavelength detector at 260 nm, Degasys DG 2410; Embase software) on a Chrom Hypersil ODS C-18 column (5 µm, 250 × 16 mm) column. Three different methods were established using binary gradients of water (A) and acetonitrile (B) and a flow rate of 10 mL/min:

Method 1: 0 to 25 min: linear from 40% B to 100% B; 25 to 35 min: isocratic 100% B.

Method 2: 0 to 20 min: linear from 30% to 40% B; 20 to 25 min: linear from 40% to 60% B; 25 to 30 min: linear from 60% to 100% B; 30 to 40 min: isocratic 100% B.

Method 3: 0 to 5 min: linear from 40% to 55% B; 5 to 25 min: isocratic 55% B; from 25 to 30 min: linear from 55% to 100% B; 30 to 50 min: isocratic 100% B.

#### 3.1.2. UHPLC/+ESI-QTOF-MS/MS

High-resolution mass determinations were performed on a Dionex Ultimate 3000 RS Liquid Chromatography System on a Dionex Acclaim RSLC 120, C18 column (2.1 × 100 mm, 2.2 µm) with a binary gradient (A: water with 0.1% formic acid; B: acetonitrile with 0.1% formic acid) at 0.8 mL/min: 0 to 9.5 min: linear from 5% B to 100% B; 9.5 to 12.5 min: isocratic 100% B; 12.5 to 12.6 min: linear from 100% B to 5% B; 12.6 to 15 min: isocratic 5% B. The injection volume was 2 µL. Eluted compounds were detected using a Dionex Ultimate DAD-3000 RS over a wavelength range of 200–400 nm and a Bruker Daltonics micrOTOF-QII time-of-flight mass spectrometer equipped with an Apollo electrospray ionization source in positive mode at 5 Hz over a mass range of *m/z* 50–1,000 using the following instrument settings: nebulizer gas nitrogen, 5 bar; dry gas nitrogen, 9 L/min, 220 °C; capillary voltage 4,500 V; end plate offset −500 V; transfer time 70 µs; collision gas nitrogen; collision energy and collision RF settings were combined to each single spectrum of 1,000 summations as follows: 250 summations with 20% base collision energy and 130 Vpp + 250 summations with 100% base collision energy and 500 Vpp + 250 summations with 20% base collision energy and 130 Vpp + 250 summations with 100% base collision energy and 500 Vpp. Base collision energy was 50 eV for precursor ions with a *m/z* less than 500 and then linearly interpolated against *m/z* up to a maximum of 70 eV for precursor ions with a *m/z* of up to 1000. Internal dataset calibration (HPC mode) was performed for each analysis using the mass spectrum of a 10 mM solution of sodium formiate in 50% isopropanol that was infused during LC re-equilibration using a divert valve equipped with a 20 µL sample loop.

Sample concentration: concentration of pure compounds: 0.1 mg/mL in methanol; concentration of crude extract: 1 mg/mL.

#### 3.1.3. NMR Spectroscopy

NMR spectra were recorded with a Varian AS 400 Mercuryplus spectrometer at room temperature in CDCl_3_ (purity 99.8%, Merck). Spectra were referenced to the CHCl_3_ solvent signal at δ 7.260 ppm.

### 3.2. Isolation Process

#### 3.2.1. Plant Material

*Achillea ptarmica* L. was cultivated at the garden of the Institute of Pharmaceutical Biology and Phytochemistry (IPBP, Münster, Germany). Aerial parts were collected at the full flowering stage in August 2011. The plant was identified by T. J. Schmidt. A voucher specimen (# TS_AchPt_01) is deposited at the IPBP herbarium. The plant material was air-dried at room temperature and powdered with an IKA MF basic mill to the riddle mesh size of 1 mm.

#### 3.2.2. Soxhlet Extraction

The powdered plant material (200 g) was exhaustively extracted with dichloromethane (1500 mL) in a Soxhlet apparatus for 12 h. The extract was evaporated to dryness under reduced pressure, yielding 6.89 g of crude extract.

#### 3.2.3. Gravity Flow Column Chromatography (CC)

The extract (6.88 g) was applied on 1.2 kg of silica gel (particle size 0.063 to 0.2 mm; Merck, column dimensions: 110 × 6 cm). The silica was equilibrated at 90:10 n-hexane/EtOAc (2,400 mL). The flow was adjusted to 1 mL/min and 10 mL of the eluate were collected per tube. A gradient with increasing amount of EtOAc was used: *n*-hexane/EtOAc 90:10 (5 L); 80:20 (7.5 L); 70:30 (6 L); 60:40 (3.5 L); 50:50 (2.5 L); 0:100 (2.5 L). Related fractions were combined after TLC control (silica gel 60 F_254_, Merck (10 × 20 cm); detection: anisaldehyde/sulfuric acid, UV 254 nm, 366 nm and daylight; elution: current solvent mixture of the CC column). The fractionation is summarized in Table 3.

**Table 3:** CC fractionation of *A. ptarmica* CH_2_Cl_2_ extract.

	Combined eluates (10 mL/tube)	Elution volume (mL)	Yield (g)	Isolated compound
Fraction I–XIV	1–1070	10,700	2.4906	
Fractions XV–XVIII containing chlorophylls	1071–1359	2890	0.3426	
Fractions XIX-XXX containing alkamides	XIX 1390–1460	1100	0.0889	
	XX 1461–1560	1000	0.3034	
	XXI 1561–1600	400	0.2738	**1**
	XXII 1601–1780	1800	0.4904	**2**
	XXIII 1781–1920	2200	0.1344	**6**
	XXIV 2001–2070	700	0.0187	
	XXV 2071–2120	500	0.0635	**3**, **4** + **5**
	XXVI 2121–2160	400	0.0196	
	XXVII 2161–2310	1500	0.1627	
	XXVIII	950	0.1017	**3**, **4** +** 5**
	XXVIX	940	0.1139	
	XXX	4920	empty	
	total	30,000	4.6042	

#### 3.2.4. Purification of the Alkamides by Preparative High Performance Liquid Chromatography (prep. HPLC)

Pellitorine (**1**) was isolated in a yield of 3.2 mg from 64.9 mg of fraction XXI using HPLC method 1. A portion of fraction XXII (34.3 mg), after purification with HPLC method I, yielded 1.7 mg of 8,9-Z-dehydropellitorine (**2**). Compound **3** (1.7 mg) and the mixture of compounds **4** and **5** (3.0 mg) were obtained from 30.8 mg of fraction XXVII and 89.2 mg of fraction XXVIII after HPLC separation using method 2. Anacycline (**6**) was purified from 86 mg of fraction XXIII using HPLC method 3 which yielded 1.3 mg of **6**. All compounds were obtained as slightly yellowish oils.

#### 3.2.5. Analytical Data

(*E,E)-2,4-decadienoic acid isobutylamide* (pellitorine, **1**) UHPLC/+ESI-QTOF MS: Rt 7.67 min, MS (*m/z*): 224.2020 [M+H]^+^; calcd. for C_14_H_26_NO^+^: 224.2009); ^1^H-NMR (400 MHz, CDCl_3_; δ (ppm), mult., *J* (Hz)): 7.18 (dd, 10, 15; H-3); 6.13 (dd, 10, 15; H-4); 6.06 (dt, 15, 6; H-5); 5.75 (d, 15, H-2); 5.48 (br t, ≈5, NH); 3.16 (dd (2H), 6, 7; H-2'); 2.14 (dt [q] (2H), ≈6, 7; H-6); 1.80 (sept, 7, H-3'); 1.41 (quint (2H), 7, 15; H-7); 1.28 (m (4H); H-8, H-9); 0.92 (d (6H), 7, H-4', 5'); 0.88 (t (3H), 7, H-10).

*(E,E,Z)-2,4,8-decatrienoic acid isobutylamide* (8,9-dehydropellitorine **2**) UHPLC/+ESI-QTOF MS: Rt 7.05 min, MS (*m/z*): 222.1855 [M+H]^+^; calcd. for C_24_H_24_NO^+^: 222.1855); ^1^H-NMR (400 MHz, CDCl_3_; δ (ppm), mult., *J* (Hz)): 7.19 (dd, 10, 15; H-3); 6.15 (dd, 10, 15; H-4); 6.07 (dt, 15, 6; H-5); 5.75 (d, 15, H-2); 5.47 (dqt, 11, 7, 1; H-9); 5.38 (dtq, 11, 7, 1; H-8); 3.16 (dd (2H), 6, 7; H-2'); 2.20 (m (4H), H-6, 7); 1.81 (sept, 7, H-3'); 1.60 (br d (3H), ≈7; H-10); 0.92 (d (6H), 7, H-4', 5').

*(E,E)-2,4-undecadien-8,10-diynoic acid isobutylamide* (**3**) UHPLC/+ESI-QTOF MS: RT 6.44 min, MS (*m/z*): 230.1541 [M+H]^+^ (calcd. for C_15_H_20_NO^+^: 230.1539); ^1^H-NMR (400 MHz, CDCl_3_; δ (ppm), mult., *J* (Hz)): 7.19 (dd, 11, 15; H-3); 6.20 (dd, 11, 15; H-4); 6.05 (dt, 15, 7; H-5); 5.80 (d, 15, H-2); 5.48 (br t, ≈5, NH); 3.17 (dd (2H), 6, 7; H-2'); 2.39 (m (4H), H-6, 7); 1.98 (s, H-11); 1.80 (sept., 7, H-3'); 0.93 (d (6H), 7, H-4', 5').

*(E,E)-2,4-undecadien-8,10-diynoic acid pideridide* (**4**) UHPLC/+ESI-QTOF MS: Rt 6.73 min, MS (*m/z*): 242.1546 (calcd. for C_16_H_20_NO^+^: 242.1539); ^1^H-NMR (400 MHz, CDCl_3_; δ (ppm), mult., *J* (Hz)): 7.18 (dd, 10, 15; H-3); 6.31 (d, 15, H-2); 6.25 (dd, 11, 15; H-4); 6.02 (dt, 15, 7; H-5); 3.61 (m (2H); H-2',6'); 3.49 (m (2H); H-2',6'); 2.39 (m (4H), H-6, 7); 1.98 (s, H-11); 1.5-1.75 (m; H-3', 4', 5').

*(E,E)-2,4-undecadien-8,10-diynoic acid phenethylamide* (**5**) UHPLC/+ESI-QTOF MS: Rt 6.91 min, MS (*m/z*): 278.1542 (calcd. for C_19_H_20_NO^+^: 278.1539); ^1^H-NMR (400 MHz, CDCl_3_; δ (ppm), mult., *J* (Hz)): 7.25-7.35 (m; aro H); 7.20 (dd, 10, 15; H-3); 6.18 (dd, 11, 15; H-4); 6.02 (dt, 15, 7; H-5); 5.73 (d, 15, H-2); 5.45 (br t, ≈5, NH); 3.61 (br dt [q] (2H), ≈7; H-2'); 2.85 (t (2H), 7; H-3'); 2.39 (m (4H), H-6, 7); 1.98 (s, H-11).

*(E,E)-2,4-tetradecadien-8,10-diynoic acid isobutylamide* (anacycline, **6**) UHPLC/+ESI-QTOF MS: Rt 7.75 min, MS (*m/z*): 272.2021 (calcd. for C_18_H_26_NO^+^: 272.2009); ^1^H-NMR (400 MHz, CDCl_3_; δ (ppm), mult., *J* (Hz)): 7.18 (dd, 10,15; H-3); 6.19 (dd, 10, 15; H-4); 6.07 (dt, 15, 7; H-5); 5.79 (d, 15, H-2); 3.17 (t (2H), 7; H-2'); 2.37 (m (4H); H-6, 7); 2.23 (t (2H), 7, H-12); 1.80 (sept., 7; H-3'); 1.55 (tq, (2H), 7, H-13); 0.99 (t (3H), 7; H-14); 0.92 (d (6H), 7; H-4', 5').

All analytical data were in full agreement with literature data [11,14].

### 3.3. In Vitro Assays and IC_50_ Determination

Tests for antiprotozoal activities were carried out using established standard protocols at the Swiss Tropical and Public Health Institute (Swiss TPH, Basel, Switzerland). The assays and the IC_50_ determinations were performed essentially as described previously [4].

The compounds used as positive controls in the various bioassays (see Table 1) were of commercial origin, with the exception of melarsoprol, which was a gift from the WHO. Their purity (generally > 95%) was specified by the manufacturers.

The purity of test compounds was assessed by UHPLC/MS and ^1^H-NMR analyses and found to be >95% in case of compounds **1** and **6**, >80% in case of compound **2**. Compounds **4** + **5** represented a mixture in a ratio of approximately 73%:27%. Compound **3** was isolated together with about 18% **4** + **5** (compare Figure 2).

## 4. Conclusions

While antiprotozoal activity has been reported for various polyacetylenes obtained from the Asteraceae and other families [2,3], such activity could be shown for the first time in this study for several alkamides with a 2*E*,4*E*-diene moiety.

Pellitorine was found the most active compound against *Pf* whose activity is sufficiently high to explain the effect of the crude extract. Since a single alkamide matching the antitrypanosomal activity and selectivity of the crude dichloromethane extract of *A. ptarmica* could not yet be isolated (8,9-*Z*-dehydropellitorine as the most active isolated compound is about three times less active than the extract), we are currently continuing these efforts in order to isolate further compounds present in the extract at lower concentrations, which might be more active. Furthermore, combinations of alkamides will be tested in order to investigate the possibility of synergistic effects.

It is also interesting to note that extracts of *E. purpurea* and *A. pyrethrum*, both known to contain a rich variety of alkamides [12,13,15], were found active against *Tbr* and *Pf*. Even though neither of them matches the activity of *A. ptarmica* against *Tbr*, we are currently in the course of isolating and testing their alkamides which will also be of value for structure-activity studies within this new class of antiprotozoal natural products.
